# Clinical and Laboratory Characteristics of an Acute Chikungunya Outbreak in Bangladesh in 2017

**DOI:** 10.4269/ajtmh.18-0636

**Published:** 2018-12-10

**Authors:** Md. Mujibur Rahman, SK. Jakaria Been Sayed, Md. Moniruzzaman, A. K. M. Humayon Kabir, Md. Uzzwal Mallik, Md. Rockyb Hasan, Abu Bakar Siddique, Md. Arman Hossain, Nazim Uddin, Md. Mehedi Hassan, Fazle Rabbi Chowdhury

**Affiliations:** 1 Department of Medicine, Dhaka Medical College, Dhaka, Bangladesh;; 2 Director General of Health Services, Dhaka, Bangladesh;; 3 Department of Internal Medicine, Bangabandhu Sheikh Mujib Medical University, Dhaka, Bangladesh;; 4 Centre for Tropical Medicine and Global Health, Nuffield Department of Medicine, University of Oxford, Oxford, United Kingdom;; 5 Mahidol Oxford Tropical Medicine Research Unit, Mahidol University, Bangkok, Thailand;; 6 Peter Medawar Building for Pathogen Research, University of Oxford, Oxford, United Kingdom

## Abstract

From April to September 2017, Bangladesh experienced a huge outbreak of acute Chikungunya virus infection in Dhaka. This series describes the clinical and laboratory features of a large number of cases (690; 399 confirmed and 291 probable) suffered during that period. This observational study was carried out at Dhaka Medical College Hospital, Bangladesh. The median age of the patients at presentation was 38 years (IQR 30–50) with a male (57.3%) predominance. Hypertension and diabetes were the most common comorbidities. The mean (±SD) duration of fever was 3.7 (±1.4) days. Other common manifestations were arthralgia (99.2%), maculopapular rash (50.2%), morning stiffness (49.7%), joint swelling (48.5%), and headache (37.6%). Cases were confirmed by anti-chikungunya IgG (173; 43.3%), IgM (165; 42.3%), and reverse transcription polymerase chain reaction (44; 11.0%). Important laboratory findings include high erythrocyte sedimentation rate (156; 22.6%), raised serum glutamic pyruvic transaminase (73; 10.5%), random blood sugar (54; 7.8%), leukopenia (72; 10.4%), thrombocytopenia (41; 5.9%), and others. The oligo-articular (453; 66.1%) variety of joint involvement was significantly more common compared with the poly-articular (237; 34.5%) variety. Commonly involved joints were the wrist (371; 54.1%), small joints of the hand (321; 46.8%), ankle (251; 36.6%), knee (240; 35.0%), and elbow (228; 33.2%). Eleven cases were found to be complicated with neurological involvement and two of them died. Another patient died due to myocarditis. Public health experts, clinicians, and policymakers could use the results of this study to construct the future strategy tackling chikungunya in Bangladesh and other epidemic countries.

## INTRODUCTION

Chikungunya fever is a viral illness caused by a single-stranded RNA virus belonging to the *Alphavirus* family.^1^ The virus is transmitted between humans through the bites of infected female, *Aedes* mosquitoes.^2^ The first documented case of chikungunya virus (CHIKV) infection in humans was in 1952–1953 at Tanganyika, East Africa, and the first outbreak occurred in Bangkok, Thailand, in 1958.^3,4^ It was then followed by a decade-long epidemic in India from 1963 to 1973 and the largest documented outbreak from 2004 to 2007 in the Indian Ocean islands.^5,6^ In December 2008, the first chikungunya outbreak was confirmed in the Rajshahi and Chapai Nawabganj districts of Bangladesh.^7^ In 2011, another outbreak occurred in the Dohar subdistrict of Dhaka where several hundred patients were exposed.^7^ Since then, it has been determined to be a potential cause of acute febrile illness in adults in Bangladesh based on anecdote and case reports.^7–12^ The classical clinical manifestation of chikungunya fever is high-grade fever, maculopapular rash, and joint pain or arthralgia.^13^ The patient can experience varied rheumatological manifestations mainly chronic and disabling arthritis of varying joints.^1,14^ Other systemic manifestations such as gastrointestinal (diarrhea, vomiting, and abdominal pain), ocular (conjunctivitis, optic neuritis, iridocyclitis, episcleritis, retinitis, and uveitis), and neurological involvement (encephalitis, myelitis, Guillain-Barre syndrome [GBS], and others) can occur.^10,15–17^

From April to September, 2017, Bangladesh experienced a huge outbreak of acute CHIKV infection in the capital city Dhaka. The objective of the present study is to describe the sociodemographic, clinical, and laboratory characteristics of the acute cases that occurred during the outbreak. The 2011 outbreak was published before from Bangladesh where only the demographic characteristics were described and the recent outbreak (2017) was reported principally emphasizing the economic impact and quality of life measurement.^7,18^ This article is the first series from Bangladesh where the detail clinical pictures of the acute cases during an outbreak is described.

## MATERIALS AND METHODS

This was an observational study carried out at the Chikungunya Clinic of Dhaka Medical College Hospital (DMCH), Dhaka, Bangladesh, from May 2017 to September 2017. This is the biggest tertiary care hospital in the country comprising 2,500 beds and receives patients from every corner of the capital and country. Patients fulfilling the inclusion criteria and clinically diagnosed as a case of chikungunya as per World Health Organization guidelines^13^ were enrolled in this study. Patients were also described as classical cases (probable and confirmed), and classical cases with neurological (tingling and numbness of extremities, altered consciousness, unconsciousness, and paraparesis) and gastro-intestinal (diarrhea and vomiting) involvement for hematological and biochemical comparison. Chikungunya virus encephalitis was confirmed either by positive polymerase chain reaction (PCR) or by serology of cerebro spinal fluid (CSF). In addition, the International Encephalitis Consortium criteria were used to classify patients according to an up-to-date definition of encephalitis.^19^ Demographic characteristics, comorbidities, history, and physical examination findings were recorded in all cases. Chikungunya virus infection was confirmed by either a reverse transcription-PCR (RT-PCR) and/or an anti-chikungunya IgM/IgG serologic test. Complete blood count, serum glutamic pyruvic transaminase (SGPT), and random blood sugar random blood sugar (RBS) were also completed in all cases. Chest X-ray posterior anterior (PA) view, electrocardiography, echocardiography, serum electrolytes level, Troponin-I, Dengue non-structural protein 1 (NS1), Dengue IgM Ab, and CSF routine examination were also completed in necessary cases.

### Case definitions.

Cases are categorized as follows:

#### Possible case.

A patient meeting clinical criteria (acute onset of fever > 38.5°C and severe arthralgia/arthritis not explained by other medical conditions).^13^

#### Probable case.

A patient meeting both the clinical and epidemiological criteria (residing or having visited epidemic areas, having reported transmission within 15 days before the onset of symptoms).^13^

#### Confirmed case.

A patient meeting the laboratory criteria, irrespective of the clinical presentation.^13^

#### Laboratory criteria.

At least one of the following tests in the acute phase^13^:

1. Virus isolation
2. Presence of viral RNA by RT-PCR
3. Presence of virus-specific IgM antibodies in a single serum sample collected in an acute or convalescent stage.
4. A 4-fold increase in IgG values in samples collected at least 3 weeks apart.

### Statistical analysis.

Data were prospectively collected by interviewing the patient or his/her legal guardian/attendant through a case record form. The study was explained to them and consent was taken. The results were expressed as a mean ± standard deviation, median with IQR, and the level of significance was expressed as *P* values unless otherwise stated. Quantitative data were presented through frequency and percentage. Student *t*-test was applied to obtain the level of significance. Statistical analysis was performed and results displayed using GraphPad Prism 7 (GraphPad Software, San Diego, CA).

### Ethical approval.

A statistician anonymized all data before analysis. The institutional ethical review committee of DMCH approved (MEU-DMC/ECC/2017/127) this study before commencement.

## RESULTS

**Figure 1.**
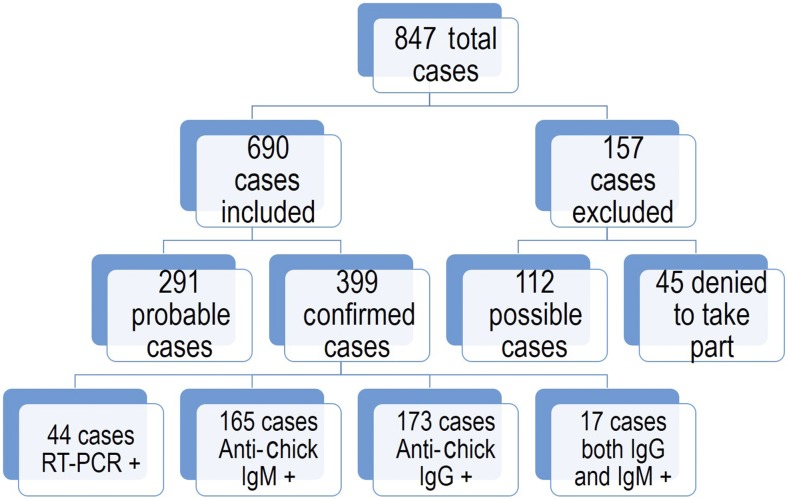
Patient’s enrollment profile during the outbreak. RT-PCR = reverse transcription polymerase chain reaction. This figure appears in color at www.ajtmh.org.

Between May and September, 2017, a total of 847 cases were enrolled in the study (Figure 1). Of this, 157 cases were excluded (112 possible cases and 45 did not provide consent to take part). In total, 690 cases were included in the study. Among them, 291 were probable cases and 399 were confirmed cases based on PCR and serological tests (Figure 1). The median age of the patients at presentation was 38 years (IQR 30–50) with a significant male (57.3%) predominance. The eldest patient was aged 96 years and the youngest was 21 years. A good proportion of the cases had hypertension (62; 8.9%) and diabetes (54; 7.8%). The other comorbidities were ischemic heart disease (IHD; 11; 1.5%), chronic kidney disease (CKD; 9; 1.3%), chronic obstructive pulmonary disease/asthma (COPD; 7; 1.0%), osteoarthritis (6; 0.8%), rheumatoid arthritis (RA; 4; 0.5%), and pregnancy (3; 0.4%). One patient had thalassemia.

The mean (±SD) duration of fever from onset to hospital consultation/admission was 3.7 (±1.4) days. Other than fever, which was present in all cases, the most common manifestations were arthralgia (685; 99.2%), maculopapular rash (347; 50.2%), morning stiffness (343; 49.7%), joint swelling (335; 48.5%), and headache (260; 37.6%) (Table 1). Some other atypical clinical features were also recorded. They were retro-orbital pain (90; 13.0%), tingling and numbness of extremities (60; 8.7%), generalized itching (24; 3.4%), diarrhea (14; 2.0%), cervical lymphadenopathy (11; 1.5%), heel pain/plantar fasciitis (11; 1.5%), altered consciousness (10; 1.4%), and others (Table 1). The mortality rate was only 0.5% (3). Among the fatalities, the first case was a 96-year-old male admitted with a short history of fever, dysuria, and disorientation. He had background CKD and his CSF was PCR positive for CHIKV. The second patient was a 55-year-old man, who presented with classical features of chikungunya fever and associated chest pain and respiratory distress. His serum IgG was high along with high troponin-I and electrocardiogram (ECG) change (ST elevation in lead II, III, and AVF). The third fatal case developed fever and paraparesis and eventually was diagnosed as GBS. He had hypertension (HTN) and diabetes mellitus (DM), and died of respiratory paralysis.

**Table 1:** Clinical characteristics and outcome of the acute Chikungunya cases (*n* = 690)

Variables	Number, %
Duration of fever (days; mean, SD)	3.7 (± 1.4)
Common clinical features*	
Arthralgia	685 (99.2)
Maculopapular rash	347 (50.2)
Morning stiffness	343 (49.7)
Joint swelling	335 (48.5)
Headache	260 (37.6)
Atypical clinical features*	
Retro-orbital pain	90 (13.0)
Tingling and numbness of extremities	60 (8.7)
Generalized itching	24 (3.4)
Diarrhea	14 (2.0)
Cervical lymphadenopathy	11 (1.5)
Heel pain (plantar fasciitis)	11 (1.5)
Altered level of consciousness	10 (1.4)
Hypotension	5 (0.7)
Others†	8 (1.1)
Mortality	
Survived	687 (99.5)
Died	3 (0.5)

* Patients have multiple variables.

† Includes aphthous-like ulceration, skin excoriation and blister, gum hypertrophy, chest pain (myocarditis), myositis, and paraparesis (Guillain-Barre syndrome).

Table 2 describes the serological, hematological, and biochemical tests performed in all cases. Of 399 confirmed cases, anti-chikungunya IgG was positive in 173 (43.3%) cases, followed by anti-chikungunya IgM in 165 (42.3%) cases and RT-PCR positive in 44 (11.0%) cases. Among the PCR positive cases, 38 were positive in blood and six were in CSF. Seventeen (4.2%) patients were found both IgG and IgM positive. Two patients were positive for concomitant CHIKV and dengue virus infection although the severity was like other classical cases. Among the hematological and biochemical tests, high erythrocyte sedimentation rate (ESR) was found in 156 (22.6%) cases. Serum glutamic pyruvic transaminase and RBS were high in 73 (10.5%) and 54 (7.8%) cases, respectively. Leukopenia and thrombocytopenia was seen in 72 (10.4%) and 41 (5.9%) cases, respectively, followed by neutropenia (35 cases; 5.0%), hyponatremia (12 cases; 1.7%), and hypokalemia (1 case; 0.1%).

**Table 2:** Diagnostic and other common positive laboratory findings of the cases

	Number; %
Positive serological tests* (*n* = 399)	
Anti-CHIK IgG	173 (43.3)
Anti-CHIK IgM	165 (41.3)
RT-PCR in Blood	38 (9.5)
RT-PCR in CSF	6 (1.5)
Both IgG and IgM positive	17 (4.2)
Hematological and biochemical tests (*n* = 690)	
High erythrocyte sedimentation rate (> 20 mm in 1^st^ hour)	156 (22.6)
High serum glutamic pyruvic transaminase (> 35 IU/L)	73 (10.5)
Leukopenia (< 4,500/mm^3^)	72 (10.4)
High random blood sugar† (> 11.1 mmol/L)	54 (7.8)
Thrombocytopenia (< 150,000/mm^3^)	41 (5.9)
Neutropenia (< 1,500/mm^3^)	35 (5.0)
Hyponatraemia (< 130 mmol/L)	12 (1.7)
Hypokalaemia (< 3.5 mmol/L)	1 (0.1)

CHIK = chikungunya; RT-PCR = reverse transcription polymerase chain reaction.

* Confirmed cases only.

† Includes few missing data.

Joint pain was present in 99.2% of cases. The oligo-articular (453; 66.1%) variety of joint involvement was significantly higher compared with the poly-articular (237; 34.5%) variety (Table 3). The most commonly involved joints were the wrist (371; 54.1%), small joints of the hand (321; 46.8%), ankle (251; 36.6%), knee (240; 35.0%), elbow (228; 33.2%), and small joints of the foot (153; 22.3%). The shoulder and hip joint were involved in 14.5% and 0.7% cases only (Table 3). Morning stiffness was present in 343 (50.0%) cases with a mean (±SD) duration of 8.6 ± 3.2 minutes.

**Table 3:** Pattern of joint involvement in acute chikungunya cases (*n* = 685)*

	Number; %
Pattern of joint involvement	
Morning stiffness (minutes; mean, SD)	343; 50.0%; 8.6 ± 3.2
Oligo-articular	453 (66.1)
Poly-articular	237 (34.5)
Type of joint involvement	
Wrist	371 (54.1)
Small joints of hand	321 (46.8)
Ankle	251 (36.6)
Knee	240 (35.0)
Elbow	228 (33.2)
Small joints of foot	153 (22.3)
Shoulder	100 (14.5)
Hip	5 (0.7)

* Patients have multiple involvements.

**Figure 2.**
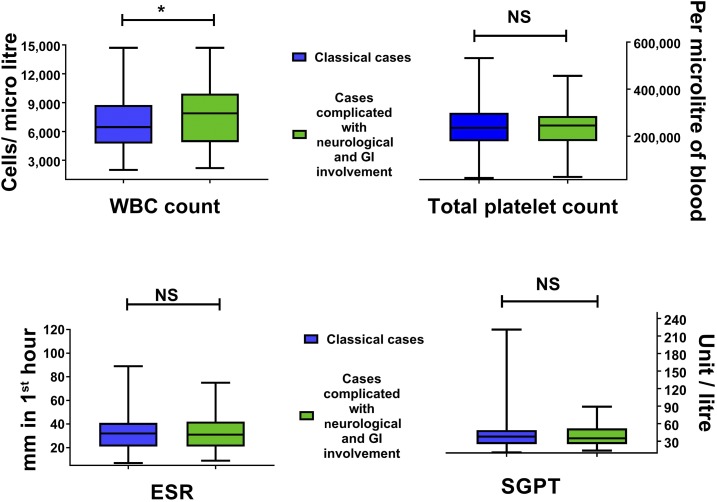
Comparison of laboratory findings between classical cases and cases complicated with neurological and GI involvement. ESR = erythrocyte sedimentation rate; SGPT = serum glutamic pyruvic transaminase. This figure appears in color at www.ajtmh.org.

Eleven acute cases were found to be complicated with neurological involvement (Table 4). Ten of them were diagnosed as encephalitis and one was GBS. All of them (except the GBS patient) presented with fever and disorientation/altered consciousness. The median (IQR) age of the patients was 63 (40–75). In total, 90.9% patients had comorbidities and the most common was hypertension (7 cases; 63.6%) and DM (6 cases; 54.5%). The SGPT level was high in seven (63.6%) cases and platelet count was low in six (54.5%) cases (Table 4). The majority (9 cases; 81.8%) were diagnosed through PCR (either CSF or blood), whereas the rest (2 cases; 18.1%) were by positive serology in blood. White blood cell (WBC) count was found to be significantly higher (*P* = 0.0143) in complicated (neurological and gastrointestinal [GI] involvement) cases compared with classical cases (Figure 2). Although no differences were seen on total platelet count (*P* = 0.9531), ESR (*P* = 0.8006) and SGPT (*P* = 0.7987) levels differed between the complicated and classical cases.

**Table 4:** Description of acute chikungunya cases with neurological involvement (*n* = 11)

Case No.	Diagnosis	Age	Clinical presentations	Comorbidities	WBC count/cu mm	Platelet count/µL	Random blood sugar mmol/L	Serum glutamic pyruvic transaminase u/L	Serological diagnosis
1	Encephalitis	85	Fever, altered consciousness	DM, HTN, ischemic heart disease	10,000	2,60,000	18	36	+PCR in CSF
2	Encephalitis	30	Fever, disorientation	–	9,525	2,05,200	4	18	+PCR in Blood
3	Encephalitis	35	Fever, altered consciousness	DM	3,360	1,48,320	6	38	Anti-CHIK IgM and IgG+
4	Encephalitis	70	Fever, disorientation, convulsion, diarrhea	DM	10,800	1,15,000	9	29	Anti-CHIK IgM and IgG+
5	Encephalitis	63	Fever, vomiting, diarrhea, disorientation	DM, HTN, CKD	6,480	2,98,000	5	89	+PCR in CSF
6	Guillain-Barre syndrome	70	Fever, paraparesis, dyspnoea	DM, HTN	2,200	1,00,000	10	60	+PCR in Blood
7	Encephalitis	52	Fever, disorientation, convulsion	HTN	5,780	2,33,300	4	46	+PCR in CSF
8	Encephalitis	96	Fever, dysuria, disorientation	CKD	14,700	27,000	8	20	+PCR in CSF
9	Encephalitis	75	Fever, disorientation	DM, HTN	8,010	1,00,000	6	60	+PCR in CSF
10	Encephalitis	56	Fever, altered consciousness	HTN	11,200	2,47,500	8	62	+PCR in Blood
11	Encephalitis	40	Fever, altered consciousness	HTN	3,980	1,42,560	10	30	+PCR in CSF

CHIK = chikungunya; CKD = chronic kidney disease; PCR = polymerase chain reaction.

## DISCUSSION

In this series, many similarities between the countries and regions were observed in the baseline demographic and epidemiological findings. However, some differences were also observed. The median age of the patients in Bangladesh was slightly lower compared with those in Sri Lanka (41 years), Indian Ocean islands (40 years), and Colombia (39 years).^20–22^ Another case series from Bangladesh investigating the same outbreak also found a lower (33.7 ± 14.8 years) mean age compared with that in ours.^18^ However, male preponderance was higher than the findings from the same countries except for Colombia. The similar male predominance was also found in Singapore.^23^ This is probably because males usually get involved with more outdoor activities compared with females in developing countries such as Bangladesh, Sri Lanka, India, and others.^24^ Moreover, access to health care for women is still restricted because of many reasons including cultural and socioeconomic factors in Bangladesh.^25–27^ Therefore, women attended less compared with males. Hypertension and diabetes were found as common comorbidities among patients and a higher percentage (12.6% and 9.4% respectively) was found in another similar series published from Bangladesh.^18^ The other common comorbidities among other studies were IHD, CKD, COPD, and others.^18,22,23^

In this series, almost all cases were manifested with arthralgia, as it is the classical presentation of chikungunya fever. The findings are identical with other studies performed in La Reunion and Gabon, although the involvement was comparatively less in Sri Lanka (71%), Singapore (87.6%), and Colombia (88%).^20–23,28^ Around half of the patients (50.2%) experienced a maculopapular rash in this series. The proportion is similar to the case series presented in La Reunion (50%), Gabon (41.8%), and Colombia (56%).^20,22,28^ By contrast, Sri Lanka reported only 11% of patients to be manifested with rash,^21^ whereas the previous case series from rural Bangladesh reported this in more than 70% of cases.^7^ Cutaneous features can be observed during the acute stage and during convalescence.^29^ Only cutaneous manifestations were described in a series consisting of 145 cases from India.^29^ They revealed pigmentary changes in 42% cases, followed by the maculopapular eruption (33%), intertriginous aphthous-like ulcers (21.3%), and generalized vesiculobullous eruptions (2.75%) as the most common forms of skin involvement.^29^ We found two patients with aphthous-like ulceration and one with blister and excoriation in this series.

Headache is another very common manifestation seen in this study. The frequency of headache was quite high in Puerto Rico (83.7%), Sri Lanka (75%), and Colombia (64%) compared with this series.^1,20,21^ Ocular pain and diarrhea occurred in 43.6% and 23.8% of cases during the acute outbreak in Puerto Rico, although it was only 13% and 2%, respectively, in this series.^1^ Digestive alterations were also quite commonly seen in Sri Lankan (11–38%) and Gabonese (32%) patients.^20,21^ Only 1.5% of patients in this series experienced adenopathy, although it was 42%, 18%, and 8% in Mexico, Sri Lanka, and Colombia, respectively.^20,21,30^ This series encountered 1.5% (11) patients suffering from plantar fasciitis in the form of heel pain. This finding is quite similar with another series described from India.^31^ One patient presented with myositis with compatible features and high creatine phosphokinase (CPK) level (23,256 IU), and it was found in another case series reported before.^1,31^ Cardiovascular features were presented in the form of hypotension and myocarditis. It is commonly seen in many other cases published from France, India, Sri Lanka, Malaysia, Colombia, Venezuela, and others.^32^ This meta-analysis found hypotension, shock and circulatory collapse, Raynaud’s phenomenon, arrhythmias, murmurs, myocarditis, dilated cardiomyopathy, congestive insufficiency, heart failure, and altered function profile as the commonly encountered cardiovascular manifestations.^32^

The Chikungunya viral arthritis usually occurs as symmetrical polyarthritis and frequently involves the peripheral joints, such as the small joints of the hands and feet, wrists, and the larger joints such as knees, ankles, and shoulders.^14,33^ Occasionally, sternoclavicular and temporomandibular joints might be involved, and hips are relatively spared.^33^ This arthritis could persist in up to 80% of cases and enter into the chronic phase.^14^ Patients in this series exhibit a similar pattern of joint involvement wherein most cases involved the wrists, small joints of hands and feet, ankles, and elbows. Only five patients complained of hip involvement. In the chronic stage, the cases might resemble some autoimmune connective tissue disease such as RA, ankylosing spondylitis, and others.^14,34^ The patients of this study are currently on a follow-up to further evaluate these chronic conditions.

During the present epidemic, several neurological manifestations have been documented such as tingling and numbness of extremities, encephalitis, and GBS. A recently published review covering 94 articles revealed encephalopathy and encephalitis, GBS, encephalomyelopathy, encephalomyeloneuropathy, and others as the most common neurological manifestation.^17^ Encephalitis and GBS/acute flaccid paralysis were most frequently reported from India during the outbreak.^35–37^ All of the neurologically involved cases (except two) were PCR positive (either CSF or blood). The reason behind these varied clinical presentations between regions is difficult to explain. The genetic and molecular diversity among different strains could be one of the possible reasons. Genetic studies during epidemics have so far revealed three evolutionary distinct genotypes, West African, East/Central/South African (ECSA), and Asian.^38^ In the Caribbean and Western Hemisphere, the virus evolved from the Asian lineage, whereas in the Indian Ocean islands and South Asia, it is of ECSA origin (Indian Ocean lineage).^38,39^ Chikungunya virus from this 2017 outbreak was recently phylogenetically analyzed and also found to be originated from the Indian Ocean clade of the ECSA genotype.^40^ It formed a novel cluster with the latest South Asian strains lacking the A226V substitution.^40^ Other potential factors for these clinical variations could be human genetic susceptibility factors, behavioral differences in exposure, inter-country differences in health-care–seeking behavior and others.

Chikungunya fever is a self-limiting disease with a very low mortality. However, deaths have been reported from different case series.^32,41^ Biochemical and hematological abnormalities, such as leukopenia, lymphopenia, thrombocytopenia, hypocalcaemia, hyponatremia, and mild-to-moderate increase in liver function tests [SGPT, serum glutamic-oxaloacetic transaminase (SGOT)] are seen with acute infections.^42^ However, these results are not specific and do not provide enough evidence to be used as a diagnostic tool. In this study, similar laboratory abnormalities were noted. In La Reunion and Sri Lanka 16.6%, 14%, and 8% of patients were found with high SGPT, respectively, which is fairly similar with this study.^22,23,42^ The frequency of cases with thrombocytopenia in two different studies in La Reunion was 9.5% and 24%.^22,42^ However, in this study, it was only 5.9%, and in Singapore, it was only 1%.^23^ Comparing classical cases and cases with neurological and GI involvement, we did not find any significant differences between total platelet count, ESR, and SGPT level. The only difference was found in the WBC count where complicated cases had a higher count compared with classical cases. The ESR, serum sodium, and potassium levels were also similar to those in other findings published before.^21,30^ A quarter (25.1%; 173) of our confirmed cases were enrolled based on 4-fold high anti-chikungunya IgG and the tests were not repeated after 3 weeks. This might be a limitation of this study. However, it is important to mention that all these cases presented with classical clinical feature, whereas an established epidemic was ongoing.

In conclusion, this series describes the clinical and laboratory features of a large number of confirmed (399) and probable (291) cases during an acute outbreak of Chikungunya infection in the capital of Bangladesh. Chikungunya fever is now spread over the five continents and is considered as a global re-emerging pathogen. Although the acute infection is self-limiting, it could turn fatal if neurological and cardiovascular involvement occurs. Those who are elderly, hypertensive, and diabetic should be managed more cautiously to avoid fatality. The Morbidity due to chronic debilitating arthritis is an important issue in terms of long-term management and increased health expenditure. The findings can be used as baseline data to plan another large cohort study during the next epidemic. This study will be pivotal in giving direction to our public health experts, clinicians, and other policymakers to construct the future strategy for effective vector control, treatment, and formulation of community and hospital-based case management guideline in Bangladesh and other epidemic countries.
